# Cell culture-based production and in vivo characterization of purely clonal defective interfering influenza virus particles

**DOI:** 10.1186/s12915-021-01020-5

**Published:** 2021-05-03

**Authors:** Marc D. Hein, Prerna Arora, Pavel Marichal-Gallardo, Michael Winkler, Yvonne Genzel, Stefan Pöhlmann, Klaus Schughart, Sascha Y. Kupke, Udo Reichl

**Affiliations:** 1 Otto-von-Guericke-University Magdeburg, Chair of Bioprocess Engineering, Magdeburg, Germany; 2 German Primate Center-Leibniz Institute for Primate Research, Infection Biology Unit, Göttingen, Germany; 3 University Göttingen, Faculty of Biology and Psychology, Göttingen, Germany; 4 Max Planck Institute for Dynamics of Complex Technical Systems, Bioprocess Engineering, Magdeburg, Germany; 5 Helmholtz Centre for Infection Research, Department of Infection Genetics, Braunschweig, Germany; 6 University of Veterinary Medicine Hannover, Hannover, Germany; 7 University of Tennessee Health Science Center, Department of Microbiology, Immunology and Biochemistry, Memphis, TN USA

**Keywords:** Influenza A virus, Antiviral, Genetically engineered MDCK cells, Defective interfering particles, DI244, Cell culture-based production, Scale up, Steric exclusion chromatography, Animal experiments

## Abstract

**Background:**

Infections with influenza A virus (IAV) cause high morbidity and mortality in humans. Additional to vaccination, antiviral drugs are a treatment option. Besides FDA-approved drugs such as oseltamivir or zanamivir, virus-derived defective interfering (DI) particles (DIPs) are considered promising new agents. IAV DIPs typically contain a large internal deletion in one of their eight genomic viral RNA (vRNA) segments. Consequently, DIPs miss the genetic information necessary for replication and can usually only propagate by co-infection with infectious standard virus (STV), compensating for their defect. In such a co-infection scenario, DIPs interfere with and suppress STV replication, which constitutes their antiviral potential.

**Results:**

In the present study, we generated a genetically engineered MDCK suspension cell line for production of a purely clonal DIP preparation that has a large deletion in its segment 1 (DI244) and is not contaminated with infectious STV as egg-derived material. First, the impact of the multiplicity of DIP (MODIP) per cell on DI244 yield was investigated in batch cultivations in shake flasks. Here, the highest interfering efficacy was observed for material produced at a MODIP of 1E**−**2 using an in vitro interference assay. Results of RT-PCR suggested that DI244 material produced was hardly contaminated with other defective particles. Next, the process was successfully transferred to a stirred tank bioreactor (500 mL working volume) with a yield of 6.0E+8 PFU/mL determined in genetically modified adherent MDCK cells. The produced material was purified and concentrated about 40-fold by membrane-based steric exclusion chromatography (SXC). The DI244 yield was 92.3% with a host cell DNA clearance of 97.1% (99.95% with nuclease digestion prior to SXC) and a total protein reduction of 97.2%. Finally, the DIP material was tested in animal experiments in D2(B6).A2G-*Mx1*^*r/r*^ mice. Mice infected with a lethal dose of IAV and treated with DIP material showed a reduced body weight loss and all animals survived.

**Conclusion:**

In summary, experiments not only demonstrated that purely clonal influenza virus DIP preparations can be obtained with high titers from animal cell cultures but confirmed the potential of cell culture-derived DIPs as an antiviral agent.

**Supplementary Information:**

The online version contains supplementary material available at 10.1186/s12915-021-01020-5.

## Background

Every year, infections with influenza A virus (IAV) result in about 300,000–650,000 deaths worldwide [1]. Occasional IAV pandemics can even cause millions of deaths, e.g., approximately 40 million deaths are attributed to the “Spanish flu” from 1918 [2, 3]. Considering the time required to develop and produce a vaccine, the availability of antivirals as a fast countermeasure seems to be indispensable for pandemic preparedness. Additionally, antivirals can also be used as a supplement to vaccination to cope with annual IAV epidemics. Antivirals currently in use are small molecules like oseltamivir and zanamivir [4–6]. However, use of these drugs is compromised by the existence of circulating resistant IAV strains [7, 8]. Thus, novel treatment modalities are clearly needed.

One such treatment modality could be the administration of defective interfering (DI) particles (DIPs) [9–13]. DIPs are virus mutants that arise naturally due to errors in the replication of the genomic viral RNA (vRNA) [14, 15]. IAV DIPs typically harbor a large central deletion in the open reading frame of one of their eight vRNA segments [16]. In addition, a DIP carrying numerous point mutations has been identified recently [17]. Due to their deletions, conventional DIPs are not capable to synthesize all full-length (FL) proteins on their own [18] but require co-infection with infectious standard virus (STV) for replication. Here, DIPs interfere with and suppress STV replication and almost exclusively non-infectious DIPs are released [19, 20]. It is speculated that this is due to faster replication of short DI vRNAs outcompeting STV for limited cellular and viral resources [16, 21, 22].

It was shown previously that administration of a specific IAV DIP containing a deletion in segment 1 (Seg1) vRNA, called DI244, resulted in an antiviral effect in animals [10–13, 23]. More specifically, treatment with DI244 containing material (produced in embryonated chicken eggs) resulted in reduced clinical symptoms in IAV-infected ferrets and protection of mice against an otherwise lethal dose of IAV [10, 23, 24]. DI244 was also reported to protect against a variety of other IAV strains [10, 23].

As DIP replication usually depends on STV co-infection, the egg-derived DIP material produced and tested in animal experiments so far has been always a mixture of DI244 and infectious STV [25]. Moreover, to eliminate potentially harmful STV in therapy, it was inactivated by UV irradiation. Yet, this also inactivated parts of the produced DIPs and consequently reduced their interfering efficacy [10]. Previously, we and others have reported methods for production of purely clonal DIP populations, overcoming the need of STV inactivation [26, 27]. These are based on genetically modified adherent and suspension cell lines expressing polymerase basic protein 2 (PB2) encoded by IAV Seg1 vRNA.

In the present study, we propose a cell culture-based production process using a suspension MDCK cell line expressing PB2 (MDCK-PB2(sus)) for production of purely clonal DI244 without STV contamination. Shake flask experiments demonstrated that the resulting DI244 yield and the interfering efficacy of the produced material strongly depended on the multiplicity of DIP (MODIP) per cell applied for infection. After scale-up to a stirred tank bioreactor (STR), the DIP material was purified using membrane-based steric exclusion chromatography (SXC). This allowed to increase its concentration about 40-fold. Finally, the DI244 material was tested in mice infected with a lethal dose of STV.

## Results

### DI244 production yields depend on MODIP

In order to produce DI244 without use of a helper virus, a purely clonal DI244 seed, generated by reverse genetics, and a genetically engineered suspension cell line expressing the viral PB2 protein, called MDCK-PB2(sus), was used. The expression of PB2 was monitored over several passages with a western blot. The cell line showed very comparable maximum specific growth rates to the parental suspension MDCK cell line (Additional file 1: Fig. S1). For process evaluation, the impact of the MODIP on DI244 yield was investigated. While earlier DI244 release and a higher yield can be expected for higher MODIPs, infections with higher virus concentrations can result in a strong de novo generation of other DI vRNAs [19], which would contaminate the DI244 product. Therefore, four MODIPs ranging from 1E−1 to 1E−4 were tested. For process monitoring, the hemagglutination assay, real-time reverse transcription qPCR (real-time RT-qPCR) and plaque assay (DI244 titer) were used.

**Fig. 1:**
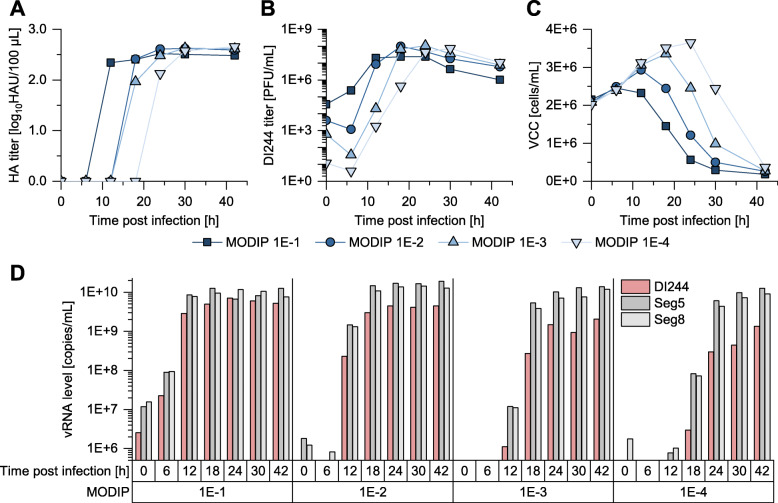
MODIP screening for production of DI244. MDCK-PB2(sus) cells were cultivated in shake flask (50 mL working volume) in Xeno™ medium. Cells were infected with a purely clonal DI244 seed virus in exponential growth phase at a VCC of 2E+6 cells/mL at MODIPs ranging from 1E−1 to 1E−4. Samples (supernatants) were analyzed for **a** HA titer, **b** DI244 titer (plaque assay in MDCK-PB2(adh) cells), **c** VCC, and **d** vRNA level of DI244, Seg5, and Seg8 (real-time RT-qPCR). Results show a single set of experiments; additional independent experiments performed for subsequent investigations (the “Interfering efficacy of DI244 material strongly depends on the MODIP used for production,” “SXC purification results in an increased interfering efficacy,” and “DI244 material shows antiviral effect in the mouse model” sections) showed comparable results (Additional file 1: Table S1)

The MODIP screening revealed that hemagglutinin (HA) and DI244 titer reached their respective maximum value earlier for higher MODIPs (Fig. 1a, b). In line with this, the viable cell concentration (VCC) decreased faster for higher MODIPs, which may be explained by an earlier onset of cell apoptosis with faster virus propagation (Fig. 1c). Maximum HA (2.62–2.65 log_10_HAU/100 μL) and DI244 titers (4.80E+7–1.08E+8 plaque forming units per mL (PFU/mL)) were comparable for all MODIPs, except for MODIP 1E−1 with slightly lower titers. For each MODIP, a decrease in DI244 titer was observed after the respective maximum was reached (Fig. 1b). This corresponds to previous findings regarding the decrease of infectious virus titers over cultivation time for wild-type IAV [28]. To analyze the interfering efficacy of biologically active DI244, material harvested at maximum DI244 titers was measured. In addition, vRNA levels (of segment 5 (Seg5), segment 8 (Seg8) and DI244) of the produced virus particles were quantified using real-time RT-qPCR. Surprisingly, maximum DI244 vRNA levels differed strongly for the different MODIPs with highest concentrations achieved for MODIPs 1E-1 and 1E-2 (Fig. 1d). In contrast, maximum vRNA levels of Seg5 and Seg8 were very comparable for all conditions.

**Fig. 2:**
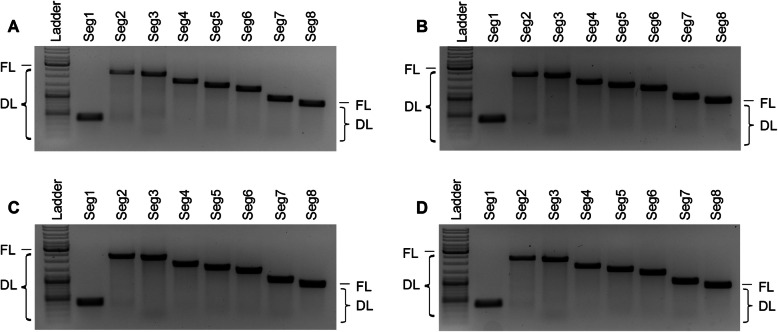
Purity of DI244 material analyzed for other defective vRNAs. DI244 produced at different MODIPs was analyzed by segment-specific reverse transcriptase-PCR for the presence of short vRNAs in Seg1–8. Results for **a** MODIP 1E−1, **b** MODIP 1E−2, **c** MODIP 1E−3, and **d** MODIP 1E−4 are shown. Signals corresponding to full-length (FL) and deleted (DL) vRNAs are indicated (FL size depends on the analyzed vRNA segment). Ladder: upper thick band 3.0 kb, middle thick band 1.0 kb, lower thick band 0.5 kb

To control contamination of the harvested material by defective vRNAs other than DI244 vRNA, a segment-specific reverse transcription-PCR (segment-specific RT-PCR) was performed (Fig. 2). However, no apparent signals indicating deleted vRNAs other than DI244 (Seg1) were observed for any MODIP on any segment.

In summary, the MODIP affected the DI244 production dynamics with earlier accumulation of DIPs at higher MODIPs. All MODIPs resulted in relatively comparable maximum virus titers. In contrast, vRNA levels of DI244 differed strongly, with lower quantities at lower MODIPs. Analysis by segment-specific reverse transcriptase-PCR did not indicate the unwanted accumulation of other vRNAs with deletions, suggesting a clean product.

### Interfering efficacy of DI244 material strongly depends on the MODIP used for production

To determine production conditions resulting in material with a high biological efficacy, an in vitro interference assay was utilized. Here, parental adherent MDCK cells were infected with STV and co-infected with the produced DI244 material, and the suppression of STV replication by DI244 assessed by comparing the released virus particles of co-infections with STV infection only. For process monitoring, hemagglutination assay, real-time RT-qPCR, and plaque assay (DI244 titer) were used.

**Fig. 3:**
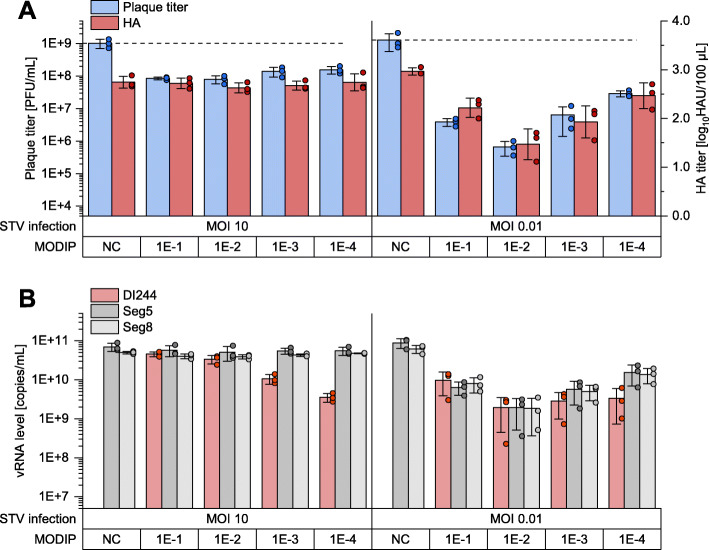
Interfering efficacy of DI244 material produced at different MODIPs. For the interference assay, parental adherent MDCK cells were infected with STV at MOIs of 10, or 0.01 and co-infected with DI244 material (125 μL), produced at a MODIP ranging from 1E−1 to 1E−4, or medium as negative control (NC). The supernatant was sampled 16 hpi (STV MOI 10) or 24 hpi (STV MOI 0.01). **a** Infectious virus titers were quantified by plaque assay (parental adherent MDCK cell). The total amount of virus particles was determined by hemagglutination assay. **b** vRNA of DI244, Seg5, and Seg8 were investigated using real-time RT-qPCR. The interference assay was performed in independent experiments (*n* = 3) using one DIP preparation for each MODIP; error bars indicate standard deviation

When STV was added at a MOI of 10, addition of DI244 material of any MODIP resulted in a plaque titer reduction of roughly one order of magnitude (*p* < 0.01, unpaired two-tailed *t* test). For cells co-infected with STV at a MOI of 0.01, larger differences between treated and untreated cells were observed (*p* < 0.05). Here, DI244 material produced at a MODIP of 1E−2 resulted in a reduction in the release of infectious STV by more than three orders of magnitude. DI244 material produced at the other MODIPs reduced the release of STV only by less than three (MODIPs 1E−1 and 1E−3) or less than two orders of magnitude (MODIP 1E−4). The difference between MODIP 1E−2 and 1E−1 was significant (*p* < 0.01), the difference between MODIP and MODIP 1E−2 and 1E−3 was not (*p* = 0.12). HA titers of samples from the interference assay showed the same trend as plaque titers (Fig. 3a). However, this reduction appeared to be less pronounced, most likely as the release of non-infectious DI244 particles themselves also contributed to the HA titer. Plaque and HA titer normalized to the corresponding NC are shown in Additional file 1: Fig. S2.

With STV at MOI 10 and addition of different DI244 preparations, all samples showed comparable vRNA levels for Seg5 and Seg8 in the interference assay (Fig. 3b). In contrast, for cells infected at MOI 0.01, strong differences for Seg5 and Seg8 vRNA levels were detected for different DI244 preparations. Here, higher vRNA level was detected for samples with a high virus titer. For all samples (STV MOIs 10 and 0.01), the vRNA levels of Seg5 and Seg8 were similar. In contrast, a lower DI244 vRNA level compared to Seg5 and Seg8 vRNA was observed for the two lowest MODIPs. This is most pronounced for cells infected with STV at a MOI of 10 treated with DI244 produced at MODIP 1E−4 (*p* < 0.005). As the segment-specific RT-PCR did not indicate accumulation of other vRNAs with a deletion (Fig. 2), the interfering effects were mainly caused by DI244.

Taken together, DI244 material produced at different MODIPs showed differences in their interfering efficacies. Material produced at a MODIP of 1E−2 showed the highest activity.

### Production in bioreactor scale results in comparable DI244 yields

**Fig. 4:**
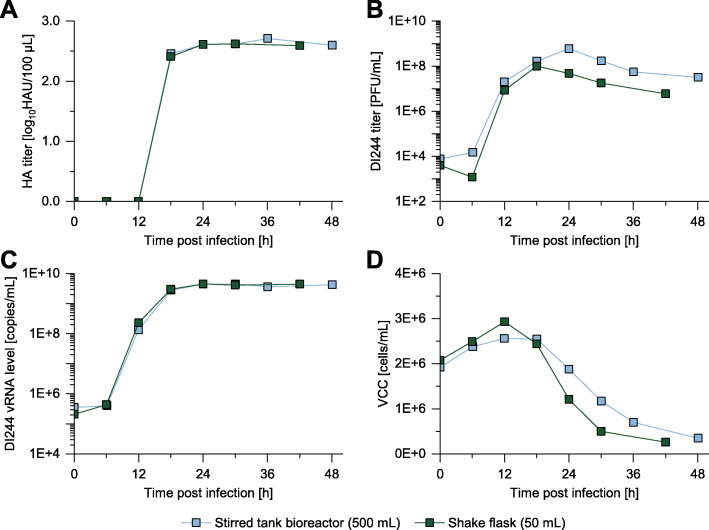
Comparison of DI244 production in a STR and a shake flask. A STR was inoculated with MDCK-PB2(sus) cells from a shake flask preculture at a VCC of 2E+6 cells/mL. Cells were infected with purely clonal DI244 seed virus at MODIP 1E−2. **a** HA titer, **b** DI244 titer (plaque assay in MDCK-PB2(adh)), **c** DI244 vRNA level (determined by real-time RT-qPCR), **d** VCC. For comparison, DI244 production in a shake flask at the same MODIP (Fig. 1) is shown

In a next step, production of DI244 material in a STR (500 mL) was investigated (Fig. 4). To allow for a comparison with shake flask cultivations, the STR was also inoculated with a VCC 2E+6 cells/mL. After the DO and pH had stabilized, cells were infected with DI244 at a MODIP of 1E−2. Note that this MODIP resulted in material showing the highest interfering efficacy for the shake flask cultivation (Fig. 3).

Despite the different production scale, aeration conditions, and pH control, very comparable results were achieved for the dynamics in the HA titer and DI244 vRNA level (Fig. 4a, c) with only minor differences for the DI244 titer and the VCC (Fig. 4b, d). Here, the shake flask cultivation showed a slightly later increase in DI244 titer (Fig. 1b) and an earlier decrease in the VCC (Fig. 1d) compared to the STR. The observed differences might be explained by the improved control of the cultivation conditions in the STR. More specifically, the pH decreased in a shake flask upon infection, potentially leading to faster cell death and increased virus degradation. In contrast, the pH was kept constant at 7.6 in the STR.

The DI244 material produced in the STR was then analyzed in the interference assay. Again, a highly comparable interfering efficacy was observed compared to DI244 material produced in the shake flask (Additional file 1: Fig. S3).

In summary, only small differences between a shake flask and a STR cultivation for DI244 production were observed, and large-scale manufacturing of DIPs in STR seems a promising option for future application in antiviral therapy.

### SXC purification results in an increased interfering efficacy

To further increase the interfering efficacy of the DI244 material harvested from shake flasks, it was purified and concentrated by membrane-based SXC. The 0.2 μm clarified virus harvest had a dsDNA concentration of 4495 ng/mL. After an enzymatic digestion, the dsDNA concentration of the sample was reduced to 78 ng/mL.

**Fig. 5:**
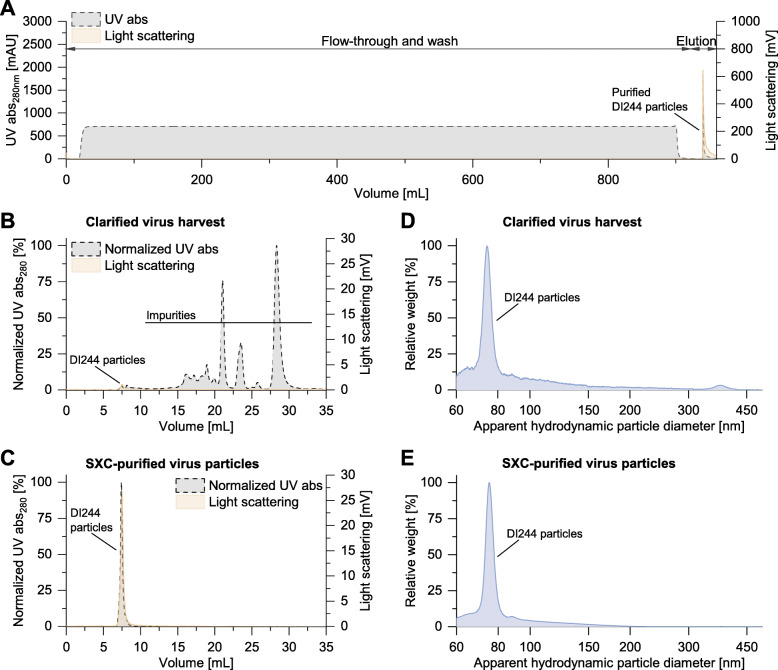
Purification of virus particles by SXC. DI244 material produced in shake flasks was purified with membrane-based SXC. **a** The presence of IAV particles in the elution step was indicated by the light scattering signal. **b**, **c** Analytical SEC fingerprints of DI244 samples **b** before and **c** after SXC purification. Based on the UV signal, the virus purity was 0.7% for the clarified virus harvest and 93.0% for the SXC-purified material. **c**, **d** Particle size distributions of DI244 material determined by DCS; **d** before and **e** after SXC purification

Around 430 mL of the clarified and digested virus harvest with a total titer of 8.5E+5 HAU were injected onto the 100 cm^2^ SXC filter device with a 1:1 in-line dilution of 16% PEG-6000, PBS 1× (Fig. 5a). The first 10 mL of the elution peak was collected, and this fraction had a total titer of 7.8E+5 HAU, representing a product yield of 92.3% and a volumetric concentration factor of around 40-fold. The HA antigen content in the SXC eluate measured by single radial immunodiffusion (SRID) assay was 16.0 μg_HA_/mL.

The collected eluate had a dsDNA concentration of 192 ng/mL (total amount 960 ng), representing a DNA clearance of 97.1% for the SXC step alone and 99.95% with respect to the undigested clarified virus harvest after enzymatic DNA digestion. The total protein concentration in the SXC eluate was 32.5 μg/mL (total amount 325.4 μg). The total protein clearance of the SXC step was 97.2% compared to the loaded material (27.2 μg/mL, total amount, 11,696 μg).

Next, the SXC eluate was dialyzed with a 300-kDa molecular mass cut-off dialysis tubing, and the collected sample was diluted around 4-fold and sterile-filtered. The dsDNA and HA antigen concentrations in this sample were 48.0 pg_dsDNA_/μL and 4.0 ng_HA_/μL, respectively.

The analytical size exclusion chromatography (SEC) fingerprints of the clarified virus harvest and the SXC-purified virus particles are shown in Fig. 5 (b and c, respectively). Here, light scattering was used to trace virus particles at a retention volume of 7.5 mL. Based on the UV signal from the SEC fingerprints, the virus purity increased from 0.7% for the clarified virus harvest to 93.0% for the SXC-purified sample. The particle size distributions of the unpurified virus harvest and the purified sample are shown as determined by differential centrifugal sedimentation (DCS) analysis in Fig. 5 (d and e, respectively). Both samples show a single monodisperse peak with an apparent hydrodynamic size of 74–75 nm.

**Fig. 6:**
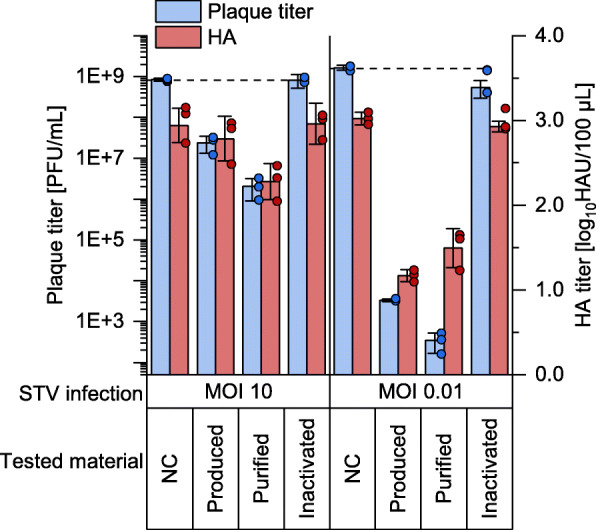
Interfering efficacy of DI244 material after SXC. Virus titers of the interference assay (see Fig. 3). Tested material was DI244 material produced in shake flask at MODIP 1E− 2, SXC purified material (as shown in Fig. 5), produced material inactivated by UV irradiation for 24 min (inactivated material) or medium as negative control (NC). Inactivated DI244 material was required as a control for animal experiments (Fig. 7). The interference assay was performed in independent experiments (*n* = 3) using one DIP preparation for each tested material. Error bars indicate standard deviation

Next, the biological activity of the purified and 4-fold diluted material was assessed in the interfering assay (Fig. 6). As expected, a strong increase in the interfering efficacy was observed (Fig. 6) with an approximately 10-fold increased reduction in the release of infectious virus particles compared the unpurified material (MOIs 10 and 0.01).

Note that parts of the produced DI244 material were UV-irradiated for 24 min as a negative control required for animal experiments (the “DI244 material shows antiviral effect in the mouse model” section). For this sample, no DI244 titer could be detected by plaque assay with MDCK-PB2(adh). Moreover, the UV-inactivated DI244 was also tested in the interference assay and no interfering activity was observed (Fig. 6).

In conclusion, SXC was used to purify and concentrate the produced material around 40-fold (10-fold after additional dilution step) with a DI244 yield of 92.3%, host cell DNA clearance of 97.1% (99.95% considering the previous nuclease digestion), and total protein clearance of 97.2%. The results of the interference assay demonstrated that efficacy was further increased and highly potent DI244 material was obtained.

### DI244 material shows antiviral effect in the mouse model

To evaluate the antiviral efficacy of DI244 in vivo, studies in a mouse infection model were performed. The tested DI244 material was produced at MODIP 1E−2 in a shake flask (active) and not purified to allow for a more stringent evaluation of toxicity. Additionally, parts of the material were UV irradiated for 24 min (inactive) as a control.

**Fig. 7:**
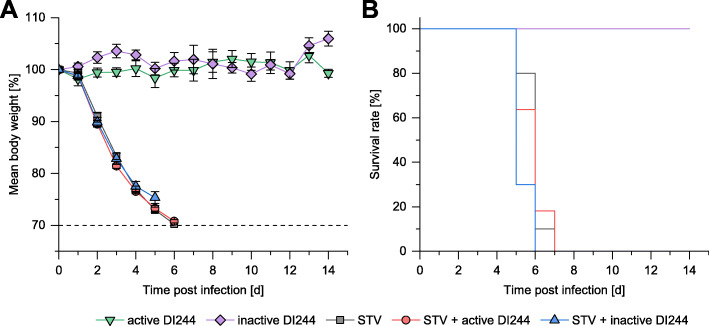
D2-*Mx1*^*−/−*^ infections with DI244 material. Female 8–12 weeks old D2-*Mx1*^*−/−*^ mice were intra-nasally infected with 20 μL solution containing 10 μL of the tested substance and 1000 FFU PR8 STV in PBS at day 0. Toxicity was tested by applying DI244 material produced in a shake flask (active, 1.5E+6 PFU per mouse; or UV-inactivated). Active DI244 (*n* = 5): D2-*Mx1*^*−/−*^ mice treated with DI244 only; inactive DI244 (*n* = 5): D2-*Mx1*^*−/−*^ mice treated with UV-inactivated DI244 only; STV (*n* = 10): D2-*Mx1*^*−/−*^ mice infected with PR8 STV; STV + active DI244 (*n* = 11): D2-*Mx1*^*−/−*^ mice infected with PR8 STV and treated with active DI244; STV + inactive DI244 (*n* = 10): D2-*Mx1*^*−/−*^ mice infected with PR8 STV and treated with UV-inactivated DI244. **a** Body weight loss curves of treated mice in percent body weight relative to day 0. Error bars indicate the standard error of the mean (SEM) for body weight changes. **b** Kaplan-Meyer survival curves. The survival rate for mice treated with active or inactive DI244 alone was 100%. Survival of mice treated only with DI244 was significantly (log rank test; *p* < 0.01) different from infected mice. Survival rates of mice treated with active DI244 versus UV-inactivated DI244 were not significantly different

First, toxicity of the DI244 was examined (Fig. 7). For this, mice were treated with DI244 material (active, 1.5E+6 PFU per mouse; or inactive) by intranasal application. Neither active DI244 nor inactivated DI244 material caused body weight loss or lethality. These results showed that DI244 alone did not cause any obvious toxic effects. Infections with STV of DBA/2JRj (D2-*Mx1*^*−/−*^) mice (lethal dose of 1000 focus forming units (FFU) PR8) resulted in death of all infected mice (Fig. 7). Co-application of active or inactivated DI244 did not rescue D2-*Mx1*^*−/−*^ mice from these lethal infections (Fig. 7). All mice lost body weight and died between 5 and 7 days post infection.

**Fig. 8:**
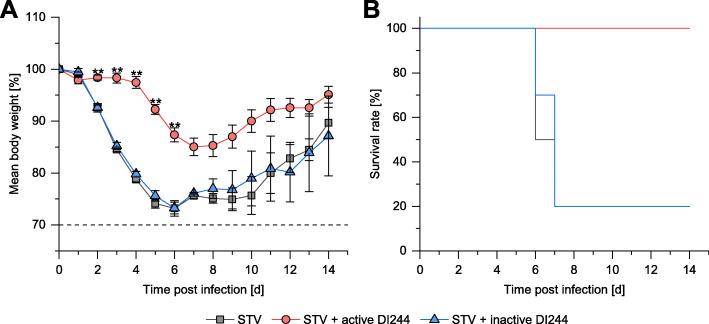
D2-*Mx1*^*r/r*^ infections with DI244 material. Female 8–12 weeks old D2-*Mx1*^*r/r*^ mice were intra-nasally infected with 20 μL solution containing 10 μL of DI244 material produced in shake flask (active, 1.5E+6 PFU per mouse; or UV-inactivated) and 1000 FFU PR8 in PBS at day 0. STV (*n* = 10): D2-*Mx1*^*r/r*^ mice infected with PR8 STV; STV + active DI244 (*n* = 12): D2-*Mx1*^*r/r*^ mice infected with PR8 STV and treated with active DI244; STV + inactive DI244 (*n* = 10): D2-*Mx1*^*r/r*^ mice infected with PR8 STV and treated with UV-inactivated DI244. **a** Body weight loss curves of treated mice in percent body weight relative to day 0. Error bars indicate the SEM for body weight changes. Statistically significant differences for body weight loss between active DI244 treated group and all other groups were assessed by unpaired two-tailed *t* test (**: *p* < 0.001). **b** Kaplan-Meyer survival curves. The survival rate for mice treated with active DI244 was significantly higher than for all other groups (log rank test; *p* < 0.0001). The survival rate for mice treated with inactive DI244 was not significant different from STV only infected mice (*p* = 0.63)

Next, D2(B6).A2G-*Mx1*^*r/r*^ (D2-*Mx1*^*r/r*^) mice [29] carrying a functional *Mx1* allele were infected with the lethal dose of 1000 FFU PR8 STV. All mice lost rapidly body weight and almost all mice died between 6 and 8 days post infection (Fig. 8). Co-application of UV-inactivated DI244 did not rescue infected D2-*Mx1*^*r/r*^ mice from body weight loss nor lethality (Fig. 8). In strong contrast, D2-*Mx1*^*r/r*^ mice co-treated with active DI244 (1.5E+6 PFU per mouse) lost much less body weight and all mice survived the infection (Fig. 8).

In conclusion, treatment of mice with DI244 material did not show any obvious toxic effects. Moreover, co-treatment of D2-*Mx1*^*r/r*^ mice with active DI244 strongly reduced body weight loss in PR8 STV infected mice and all mice survived whereas control mice treated with inactivated DI244 strongly lost body weight and died. These results clearly demonstrate the antiviral potential of DI244.

## Discussion

In this study, a cell culture-based production process for purely clonal DI244 particles without STV contamination was established. The production process was scaled up from shake flask to a STR with a working volume of 500 mL. The produced material showed high interfering efficacies in an in vitro interference assay, which was further improved after membrane-based SXC purification. Animal trials performed with unclarified shake flask harvests passed toxicity testing. Finally, mice infected with a lethal dose of IAV could be rescued by co-treatment with this DI244 material, demonstrating its antiviral potential.

### Advantages of DIPs over currently used small molecule antivirals

The administration of DIPs to prevent IAV infection might have several benefits. Specifically, DIPs show a fast mode of action, as their protective ability does not depend on the adaptive immune system, which can take up to 2–3 weeks to establish full protection in the case of vaccine administration. Because of their mode of action, DIPs could be used either prophylactically or even therapeutically [10]. It was shown by Dimmock and Easton that egg-derived and UV-inactivated DI244 material administered 7 days before infection still protected mice from a lethal dose of IAV [25]. Moreover, mice infected with a lethal dose of IAV and treated with DI244 24 h after the challenge survived [10]. Partial protection was observed, when DIPs were administered 48 h after the infection [10]. Furthermore, an antiviral effect of DI244 against a variety of IAV strains was shown [10, 23], including pandemic and highly pathogenic avian strains [30]. This suggests that DIPs, in contrast to currently used vaccines, might have the potential to act universally against IAV [25]. Additionally, an antiviral effect against influenza B and pneumovirus was demonstrated [11, 31]. This might be explained by an unspecific protection due to an induction of the innate immune response. Currently, IAV vaccines require annual adaptation to seasonal circulating strains, including accurate prediction, time-consuming generation of a seed virus, and egg or cell culture-based production. In contrast, DIP production in suspension cells would allow manufacturing a high number of doses of an antiviral drug to be better prepared for the next IAV pandemics.

Mice infected with a lethal dose of STV and treated with DI244 did not show symptoms of disease, but still developed an immunity to the pathogenic STV [10]. Here, it was speculated that DIP co-treatment results in the release of non-infectious particles carrying the surface proteins of the pathogenic virus and therefore acts like a live attenuated vaccine [25]. In line with this, it was shown that compared to an untreated control group, DI244 co-treatment did not influence the amount of specific IAV antibodies produced in ferrets. In contrast, reduced antibody titers were detected in oseltamivir treated ferrets [23], emphasizing potential advantages of DIPs over conventional antivirals. Furthermore, resistance against the small molecule antivirals oseltamivir and zanamivir has already been reported [4–6], whereas it is highly unlikely that resistance arises against DIPs: the RNA-dependent RNA polymerase complex (comprising the polymerase subunits PB2, PB1, and PA proteins) would need to mutate to not recognize and replicate the DI vRNA anymore [25]. However, the same polymerase complex replicates all eight vRNA segments [32–34]. Thus, in addition to mutation of the viral polymerase complex, it would be necessary that mutations of the polymerase recognition sequences of all eight STV vRNA segments arise simultaneously. Only under these circumstances could STV replication without DIP replication take place [25]. The probability that this happens is extremely low and was previously estimated to be around 1E−45 [25]. In conclusion, DIPs with their unique antiviral mechanism are very interesting candidates for prophylactic and therapeutic treatments showing advantages over currently used small molecule antivirals.

### Advantages and opportunities of a cell culture-based production processes

The cell culture-based production process established has several advantages over the previously reported egg-based process [10]. First, it has improved sterility, scalability, and flexibility. Second, it allows for comprehensive monitoring, and tight process control enables a reproducible product quality [19, 20, 35, 36]. Additionally, genetically modified cells can be used to allow production of purely clonal DIP preparations [26, 27], which completely overcomes the necessity of STV inactivation. Previously, UV light was used to disrupt the STV vRNA [10, 36] by introducing photodimeric lesions [37] or unspecific chain breaks [38, 39]. Here, it was speculated that larger STV vRNA (~ 2.0 kb) should be faster inactivated than the rather small DIP vRNA (~ 0.4 kb), as the probability of damaging photoreactions is higher for the STV vRNA [10]. However, it was shown recently that also the DIP vRNA is damaged by UV light, resulting in a decreasing interfering efficacy over UV inactivation time [40]. Moreover, UV inactivation was also used in the current study to generate a negative control, which did not show any interfering efficacy in the interference assay (Fig. 6) or in animal experiments (Figs. 7 and 8). In contrast, the interfering efficacy of the purely produced DIP material was maintained at a very high level. Using our approach, concerns regarding biosafety, i.e., the risk of residual infectious STV due to incomplete inactivation after UV treatment can be avoided.

In principle, the genetically modified MDCK-PB2(sus) may be used universally for cell-culture based production of any IAV Seg1 DIP [26]. The interfering efficacy of a DIP seems to be affected by many factors including genome length, genome sequence, and breaking point [16, 41–43]. Therefore, other DIPs could have a higher interfering efficacy or offer additional advantages over DI244. The generation of genetically modified cell lines expressing another viral protein, e.g., the viral PB1 or PA protein, would also allow production of purely clonal Seg2 or Seg3 DIPs. These segments are of special interest, as deletions in Seg1–3 are most frequently observed [16, 44]. Here, it was hypothesized that DIPs originated from polymerase genes (Seg1–3) may have advantages over DIPs originating from structural genes (Seg4–8) [22]. For the generation of a purely clonal DIP seed virus, the reverse genetics approach reported earlier represents a universal platform [26].

Lastly, the separation principle of the SXC allows purification of any IAV strain using a single recipe [45]. Therefore, the established platform covering cell line generation, seed virus generation, DIP production, and DIP purification allows to quickly produce a wide range of DIP candidates for testing in an animal model for further use as an antiviral.

### The MODIP affected the incorporation of DI244 vRNA in the produced virus particles

In the present study, Seg5 and Seg8 vRNA levels (considered representative for all STV vRNA segments) were approximately equimolar for each MODIP and each sampling time point. In contrast, DI244 vRNA levels were always lower. This might suggest that Seg5 and Seg8 vRNA were present in every virus particle, whereas the DI244 vRNA was absent in some virus particles. Usually, virus assembly and budding were considered a well-organized process, where each of the eight vRNAs is incorporated in the produced virus particle only once [46, 47]. This is facilitated by the packaging sequence, present at the 3′ and 5′ end of each vRNA segment [48, 49]. Nevertheless, depending on the strain, up to 20% of produced viruses do not package at least one vRNA segment [50]. This results in the generation of semi-infectious particles [51]. Naturally occurring virus mutants which completely miss several vRNA segments have also been observed [17].

The MDCK-PB2(sus) cell line used here expressed the viral PB2 protein, encoded by Seg1 vRNA. With the cell line providing the missing PB2 protein, the virus propagation theoretically does not require an intact Seg1 vRNA. Concurrently, also the deleted Seg1 vRNA from DI244 is not essential for replication. Therefore, the MDCK-PB2(sus) cell line might not only allow production of purely clonal Seg1 DIPs, but also propagation of viruses with only seven segments, completely missing the Seg1 vRNA. This might also explain the lower level of DI244 vRNA in the produced virus particles. Furthermore, DI244 vRNA levels decreased with lower MODIPs. A possible explanation may be that higher MODIPs result in overall more co-infections and therefore a higher probability that all eight segments are present in an infected cell. Here, most produced viruses would incorporate all eight segments. In contrast, in a low MODIP scenario, the likelihood for single-hit infections is increased drastically. Under this condition, cells may occasionally be infected by a virus without DI244 vRNA. Consequentially, those cells could only produce viruses also missing DI244 vRNAs. Additionally, the produced 7-segmented viruses will further accumulate in subsequent infection waves (which are characteristic for low MODIPs).

The lack of DI244 vRNA could also explain the observed differences between plaque and interference assay. More specifically, MODIPs of 1E−2 to 1E−4 resulted in very comparable DI244 titers. In contrast, large differences in the interfering efficacy were observed, where material produced with lower MODIPs induced a less pronounced titer reduction. The DI244 titer was evaluated in a plaque assay with MDCK-PB2(adh) cells. Here, virus particles without incorporated DI244 vRNA could still replicate and would therefore contribute to the DI244 titer. On the other hand, particles without DI244 vRNA would not interfere with STV replication. Therefore, these particles would not contribute to the interfering efficacy determined in the interference assay. Consequentially, material produced at a MODIP of 1E−2, showing the highest DI244 vRNA level (for MODIP 1E−2 to 1E−4) resulted in the most pronounced titer reduction. DIP material produced at a MODIP of 1E−1 showed comparable vRNA levels, but lower DI244 titers. A possible explanation could be that the interfering efficacy of this material is lower due to a faster onset of DI244 production, resulting in an earlier onset of degradation of biologically active virus particles and a reduced number of virions carrying DI244 vRNA to the cells. Another explanation for the observed reduction of DI244 and HA titers of material produced at a MODIP of 1E−1 might be a self-interference of DIPs at high MODIPs, as reported by other groups [52, 53].

The mechanism proposed here, where DI vRNAs might not be efficiently incorporated in the produced virus particles, when produced with a complementing cell line, would have implications for the DIP production process. The risk that high amounts of virus particles without any therapeutic effect might be produced, especially at lower MODIPs (usually used for cell culture-based viral vaccine production) is of particular importance. Therefore, optimization of the MODIP seems crucial for the establishment of a production process as it might drastically affect the quality of DIP harvests.

### Purification of DI244 harvests

The product yield of the SXC purification step measured by hemagglutination assay was 92.3%. This is consistent with previous results for downstream processing of IAV [54], as was the concentration of the HA antigen in the SXC eluate of this work (16.0 μg_HA_/mL). The clearance of host cell DNA was 97.1%, and adding a DNA digestion step prior to SXC increased the DNA clearance to 99.95%. The total protein clearance was 97.2%, which is also consistent with previously reported data [54].

The dsDNA concentration of the clarified virus harvest in this work was 4495 ng/mL compared to around 4300 ng/mL from a similar process for IAV production using SMIF8 chemically defined medium [54]. With a dsDNA concentration of 192 ng/mL in the eluate, the estimated dsDNA concentration of this material was 0.05 ng/μL and therefore about 100-fold lower than that of the unpurified material (4.6 ng/μL) administered to mice for toxicity testing. A further reduction in the amount of DNA can be achieved, for instance with optimizations in cell culture that could reduce the total burden of DNA introduced into the purification train, a longer DNA enzymatic digestion, or with additional polishing steps, such as pseudo-affinity chromatography with sulfated membrane adsorbers [55] or ion exchange chromatography.

Another interesting topic would be the separation of active DI244 particles from 7-segmented viruses, discussed in the previous section. The difference in the nucleotide cargo between the two virus populations might result in an isoelectric point difference that could be exploited for their chromatographic separation by isoelectric focusing [56, 57] or even ion exchange. These options provide alternatives for future work.

### Evaluation of DI244 interfering efficacy in animal and in vitro models

Administration of DI244 alone did not induce body weight loss nor result in a decreased survival rate and therefore did not appear to be highly toxic. To further elaborate potential toxicity of DI224 administration, histopathology or blood chemistry of mice could be performed [58]. Co-treatment with DI244 did not show a positive impact on body weight loss or survival rate in PR8 (H1N1) STV infected D2-*Mx1*^*−/−*^ mice. In strong contrast, infected D2-*Mx1*^*r/r*^ mice treated with DI244 showed a reduced body weight loss and all animals survived the infection.

MX1 represents an interferon-induced protein, which binds to the ribonucleo-protein particles of IAV and thereby inhibits viral replication [59, 60]. The protective activity of MX1 against IAV was originally discovered in A2G mice that carry a wild-type *Mx1* allele [59, 61]. However, most laboratory strains, including the most commonly used strain, C57BL/6, do not carry a functional *Mx1* allele [59, 62]. These common laboratory mice express *Mx1* transcripts with a deletion or nonsense mutation in the open reading frame resulting in a non-functional protein [59, 62]. The wild type functional *Mx1* allele has been transferred from A2G to C57BL/6 mice [63] to generate strain B6.A2G-*Mx1*^*r/r*^ (B6-*Mx1*^*r/r*^). B6-*Mx1*^*r/r*^ mice are per se highly resistant against infections with IAV [63, 64]. Since these mice scarcely show clinical symptoms and mortality, they do not allow testing of anti-viral treatments.

Humans carry a functional *MX1* allele, but are still susceptible to IAV infections. Therefore, we generated a mouse model that better mimics the human situation. We introduced the wild type *Mx1* allele into DBA/2 mice, resulting in mouse strain D2-*Mx1*^*r/r*^, which now expresses a fully functional MX1 protein. In contrast to B6-*Mx1*^*r/r*^ mice, D2-*Mx1*^*r/r*^ mice are still highly susceptible to IAV infections. However, after pretreatment with interferon, D2-*Mx1*^*r/r*^ become resistant to IAV infections demonstrating that in these mice a fully functional and protective MX1 protein can be produced. We previously [29] have described the detailed characterization of the D2-*Mx1*^*r/r*^ model. Thus, our D2-*Mx1*^*r/r*^ IAV infection model which has been used here for the DI244 functional studies represents an ideal system that better reflects the human situation and allows testing of antiviral treatments in the context of a fully functional *Mx1* allele.

The infection experiments with the different mouse models demonstrate the importance of a functional innate immune response for the antiviral effect of DIPs in vivo. Here, we hypothesize that DI244 in mice inhibits viral replication, as we have demonstrated in our in vitro studies, but in addition induces interferon, which subsequently activates the highly protective functional *Mx1* gene in D2-*Mx1*^*r/r*^ mice. Both effects will lead to lower viral loads in the lung, the rapid induction of a potent innate immune response, and protection from a lethal outcome. Virus-specific antibodies and cytotoxic T cells typically start to appear at 7 days post infection and are fully mounted after 14 days. Thus, an adaptive immune response would come too late in a primary infection to protect against a severe outcome during the first week. We thus conclude that in this model, DI244 does not primarily have a vaccination effect. However, mice treated with DIPs will survive the infection, and in these mice, an adaptive immune response will be mounted. Such an adaptive immune response will protect against a secondary infection [10]. Therefore, DIP treatment will not only be beneficial for protection against severe disease in the early phase of a primary IAV infection but also contribute to mounting a long-lasting protective immunity. Here, it would also be of interest to investigate the impact of the time of DIP application (e.g., a few days before or after challenge virus administration).

The in vitro interference assay used here was carried out with MDCK cells, which express a canine MX1 lacking activity against the human IAV strain PR8 [65]. Furthermore, trypsin added to the medium used in the interference assay degrades the secreted interferon [66]. Therefore, the interfering effects observed in the in vitro assay are most likely explained by DIPs interfering with the replication of the STV, rather than induction of the innate immune response. In order to understand better the contribution of the innate immune response to the interference of DIPs in vitro, additional experiments are necessary. For example, the interference assay could be carried out with a human cell line carrying a functional MX1 (e.g., A549 or HEK293 cells). Additionally, to avoid interferon degradation by trypsin, the virus strain A/WSN/33, which does not rely on trypsin addition for its propagation [67], could be used and is topic of ongoing research.

Finally, it would be desirable to conduct infection experiments in ferrets, as they are susceptible to human IAV and air-borne virus transmission [68–70]. In a next step, infection experiments in macaques could be carried out, as their clinical symptoms closely resemble those found in humans [71]. Trials in both animals would represent a significant step towards studies in humans to demonstrate the protective effect of DIPs and the use of DIP preparations as antiviral drugs. Here, it would also be of interest to investigate the impact of the time of DIP application (e.g., a few days before or after challenge virus administration).

## Conclusion

In this proof-of-principle study, we established a cell culture-based production and purification platform for generation of purely clonal Seg1 DIP material, which does not require inactivation. The proposed platform could be used to quickly produce new DIP candidates for testing in an animal model. In addition, it allows fast scale up for manufacturing of a high number of doses if required. The produced DI244 material showed strong antiviral effects in animal experiments. As far as we are aware, this is the first time purely clonal DIP material was produced at bioreactor scale. Additionally, it was the first time purely clonal DIP material that does not require UV inactivation positively demonstrated its antiviral potential in an animal model.

## Methods

### Cells and viruses

For production of non-infectious DIPs (DI244 containing a large internal deletion on Seg1 vRNA), a suspension MDCK cell line adapted to growth in a chemically defined Xeno™ medium was used [72]. Next, this suspension cell line was genetically modified by retroviral transduction as described before for adherent MDCK cells [26], to stably express the viral PB2 protein and a puromycin resistance gene as selective marker. Briefly, a cell suspension of 1.0E+6 cells/mL was seeded in shake flasks in 10 mL chemically defined Xeno medium. Simultaneously, 10 mL of supernatant containing MLV particles was added per flask. In parallel, non-transduced suspension cells were supplemented with 10 mL Dulbecco’s Modified Eagle Medium containing 10% fetal bovine serum (FBS, Gibco), penicillin (100 IU/mL), and streptomycin (100 μg/mL) to maintain similar medium condition. Next day, cells were centrifuged at 300×*g* for 5 min and resuspended with 20 mL fresh chemically defined medium. Two days post transduction, cell suspension was counted and supplemented with 0.5 μg/mL puromycin. In the following, the generated cell line is called MDCK-PB2(sus).

MDCK-PB2(sus) cells were cultivated either in (i) a shake flask with 50 mL working volume (125 mL baffled polycarbonate Erlenmeyer Flask, Thermo Fisher Scientific, 4116-0125) at 37 °C, 5% CO_2_, and 185 rpm (Multitron Pro, Infors HT; 50 mm shaking orbit) or (ii) a STR with a working volume of 500 mL (DASGIP® Parallel Bioreactor System, Eppendorf AG, 76DG04CCBB) at 37 °C, ≥ 40% O_2_, pH 7.6, and 150 rpm (marine impeller). For cultivation of parental adherent MDCK cells (ECACC, No. 84121903) or adherent MDCK cells expressing PB2 [26], hereafter called MDCK-PB2(adh), Glasgow minimum essential medium (GMEM) (Thermo Fisher Scientific, #22100093) containing 1% peptone and 10% fetal bovine serum (cultivation medium) was used (37 °C, 5% CO_2_). For MDCK-PB2(adh), 1.5 μg/mL puromycin (Thermo Fisher Scientific, #A1113803) were added to the cultivation medium. For seed train generation of suspension cells, 0.5 μg/mL puromycin was added. All infection studies were performed without puromycin addition.

Purely clonal DI244 virus was generated using an eight-plasmid DNA transfection system [73], MDCK-PB2(adh), and 293 T cells expressing PB2 [26]. The virus was expanded in MDCK-PB2(sus) cells to yield a seed virus with a DI244 titer of 1.01E+8 DIPs/mL (assayed in MDCK-PB2(adh) cells, see the “Virus quantification assays” section). For STV infections, the H1N1 influenza virus A/Puerto Rico/8/34 (PR8) from the Robert Koch Institute Germany (1.1E+9 TCID_50_/mL) was used. Multiplicity of infection (MOI) was calculated based on the fifty-percent tissue culture infection dose (TCID_50_) titer [74]. For infection of MDCK-PB2 cells, MODIP was calculated based on the DI244 titer.

### Cell culture-based production of DI244

MDCK-PB2(sus), cultivated in shake flasks in exponential growth phase, were centrifuged (300×*g*, 5 min, room temperature) to allow for media exchange. Next, cells were transferred to an STR or shake flask at a VCC of 2E+6 cells/mL (with fresh media). For subsequent infection, trypsin (Thermo Fisher Scientific, #27250-018) was added (20 U/mL final activity) and DI244 seed virus at the indicated MODIP. The VCC was measured with a cell counter (Vi-CELL XR, Beckman Coulter, #731050) with a relative standard deviation (RSD) of ≤ 6%. For some analytical procedures described below, a small volume of cell suspension was centrifuged (3000×*g*, 10 min, 4 °C) and aliquots of the cell-free supernatants stored at − 80 °C until further analysis.

### UV-irradiation

UV irradiation was utilized to inactivate the produced DI244 material (required as a control for animal experiments). Treatments were carried out in a laminar hood (Thermo Fisher Scientific, Safe 2020) to ensure sterility. For this, the material was transferred into an open tray (250 cm^2^ surface area) for direct exposure using the UV lamps in the side walls of the laminar hood for 24 min. The tray was shaken continuously using a mixer (Duomax 1030, 543-32205-00 Heidolph, Schwabach, Germany) ensuring a thin film layer (approximately 2 mm) and a homogenous inactivation. Afterwards, parental adherent MDCK cells were infected with purely clonal DI244 harvests and inactivated DI244 material. For both DI244 preparations, no STV replication could be detected.

### Downstream processing of DI244 material

To increase its interfering efficacy and to reduce the level of contaminating protein and DNA, the harvested DI244 material was purified by membrane-based SXC.

#### Sample preparation before capture chromatography

DI244 material produced in shake flasks was clarified by a series of successive microfiltration steps using regenerated cellulose filter discs with a diameter of 5 cm and pore sizes of 1.0 μm, 0.45 μm, and 0.2 μm (GE Healthcare; Uppsala, Sweden) fitted to a vacuum-operated reusable bottle top device (# 528199-325; VWR; Radnor, USA). The sample volume after the final 0.2 μm filtration step is named “clarified virus harvest” hereafter.

The clarified virus harvest was treated with an unspecific nuclease (Denarase®, named “Denarase” hereafter, #2DN100KU99; Sartorius Stedim Biotech; Göttingen, Germany) to digest the host cell DNA. For this, the clarified virus harvest was supplemented with magnesium chloride (#M8266-1KG; Sigma-Aldrich Chemie GmbH; Munich, Germany) to a final concentration of 2 mM and 50 U/mL of Denarase. The sample was incubated (4.5 h, room temperature) under mixing with a magnetic stirrer at 250 rpm.

#### Chromatographic purification

For all chromatography experiments, an ÄKTA Pure 25 (GE Healthcare; Uppsala, Sweden) liquid chromatography system was used at room temperature and controlled by the software UNICORN v6.3 was used. The UV absorbance was monitored at 280 nm and virus particles were monitored with a NICOMPTM 380 (Particle Sizing Systems; Santa Barbara, USA) submicron particle analyzer at 632.8 nm.

Membrane-based SXC [54] was used for virus purification. The SXC device consisted of a stack of 1.0 μm regenerated cellulose membranes (#10410014; GE Healthcare; Uppsala, Sweden) (20 layers; 100 cm^2^ total surface area with a column volume of 1 mL) fitted into a stainless steel filter housing (25 mm). The pressure limit at the inlet of the column was set to 2.00 MPa. The flow rates used were 5–10 mL/min.

SXC purification was performed in bind-elute mode. In short, (A) Equilibration: after a washing step of 10 column volumes of water, the column was equilibrated with 10 column volumes of 8% PEG-6000, PBS. (B) Sample injection: the Denarase-treated sample was mixed in-line in a 1:1 ratio with a stock solution of PEG-6000 (#81260-5KG; Sigma-Aldrich Chemie GmbH; Munich, Germany) and PBS to achieve a final concentration of 8% PEG-6000. After sample injection (10 mL/min), a wash step (10 mL/min) followed with 8% PEG-6000, PBS until baseline UV absorbance was achieved. (C) Elution: virus particles were eluted using 20 column volumes of PBS (5 mL/min).

An analytical SEC with a packed-bed Superdex 200 Increase 10/300 GL (# 17517501; GE Healthcare; Uppsala, Sweden) column was conducted. The injection volumes were 100–500 μL with a flow rate of 0.75 mL/min.

#### Formulation

The SXC eluate was dialyzed against PBS (overnight, 4 °C) with a 300-kDa molecular mass cut-off dialysis tubing (16 × 10 mm, width × diameter) made of a cellulose ester (#GZ-02890-77; Spectra Por) with a sample to buffer ratio of 1:1000. First, the collected sample was spiked with sorbitol to a final concentration of 4%. Next, the sample was sterile-filtered (0.2 μm cellulose acetate syringe filter (#16534----------K; Sartorius Stedim Biotech; Göttingen, Germany). The final DI244 material was stored at − 80 °C until evaluation in in vitro assays or animal experiments.

### Mouse experiments

#### Mouse model

D2-*Mx1*^*−/−*^ mice carrying a non-functional *Mx1* gene were obtained from Janvier (France). D2-*Mx1*^*r/r*^ carrying a functional *Mx1* gene was generated in our laboratory by backcrossing DBA/2JRj mice for 10 generations onto congenic B6.A2G-*Mx1*^*r/r*^ mice as described previously [29]. All mice were maintained under specific pathogen free conditions at the Central Animal Facilities of the HZI, Braunschweig.

#### Infection of mice

The production of a PR8 STV used for mouse infections and titration of FFU was performed as described previously [58, 75]. Mice (female, 8–12 weeks old) were anesthetized by intra-peritoneal injection of ketamine-xylazine solution (5 mg/mL ketamine, WDT, Garbsen; 1 mg/ml xylazine, CP Pharma, Burgdorf; in sterile 0.9% NaCl, WDT, Garbsen) with a dose adjusted to the individual body weight (200 μL/20 g body weight). Mice were treated with 20 μL PBS solution containing 10 μL of DI244 material and 1000 FFU of PR8 by intranasal application. Subsequently, body weight and survival were monitored for 14 days. In addition to mice that were found dead, mice with a body weight loss of more than 30% of the starting body weight were euthanized and recorded as dead.

### Interference assay

The produced DI244 material was evaluated using an interference assay [17]. Here, to assess the interfering efficacy, the reduction in the release of infectious virions upon co-infection with STV and DI244 is compared to an infection with STV only. For infection, parental adherent MDCK cells (1E+6 cells per well) cultivated in 6-well plates were used. Cells were washed with phosphate buffered saline (PBS). Next, cells were either infected with only STV (PR8) at a MOI of 10 or 0.001 or co-infected with STV and a fixed volume of 125 μL of DI244 material. Infections were conducted in 250 μL infection medium (GMEM containing 1% peptone and 5 U/mL trypsin). After 1 h of incubation (37 °C, 5% CO_2_), the inoculum was removed and cells were washed with PBS. Next, 2 mL of infection medium were added and cells were further incubated (37 °C, 5% CO_2_). Infections were incubated for 16 h or 24 h, depending on the MOI of STV added (10 or 0.001, respectively). Supernatants were centrifuged (3000×*g*, 10 min, 4 °C), and aliquots were stored at − 80 °C until further analysis.

### Virus quantification assays

For quantification of the total virus particle titer, a hemagglutination assay was used, as described in [76]. Here, the HA titer can be either reported as log_10_HA units/100 μL (log_10_HAU/100 μL) or as HAU/100 μL and the total amount of HAU in a virus preparation can be calculated. The RSD for technical triplicates was ≤ 13.3%. For quantification of infectious virus titers, a plaque assay was used. For this, parental adherent MDCK cells in 6-well plates were utilized. Cells were washed with PBS. Next, each 250 μL of a serial 10-fold dilution series (of each sample) were used to infect the cells for 1 h (37 °C, 5% CO_2_). The supernatant was removed and cells were overlaid with 1% agar in infection medium (GMEM containing 1% peptone and 5 U/mL trypsin). Cells were then incubated for 4 days (37 °C, 5% CO_2_) until staining. Ice-cold methanol was used for fixation, and cells were stained using a 0.2% crystal violet solution. Finally, light microscopy was used to determine the plaque forming units per mL. For quantification of samples containing only DI244, the same plaque assay was used but with MDCK-PB2(adh) cells. Statistical differences were determined by unpaired two-tailed *t* test. The RSD of technical duplicates was ≤ 43.8%.

Additionally, the HA antigen content in the purified DI244 material (see Section “Downstream processing of DI244 material”) was quantified by a SRID assay as reported before [54]. The purified virus particles were dialyzed overnight at 4 °C using a 300-kDa molecular mass cut-off dialysis tubing (16 × 10 mm, width × diameter) made of a cellulose ester (#GZ-02890-77; Spectra Por) with a sample to buffer ratio of 1:1000. Dialyzed samples were spiked with sucrose as a cryoprotectant to a final concentration of 1% and lyophilized afterwards. The lyophilized samples were resuspended by adjusting the HA content of the samples (around 6200 HAU per replicate) to the HA content of an in-house reference standard as previously reported by [77]. The assay was setup in a 1% agarose gel with a 7 × 7 diffusion matrix containing 64 μg anti-A/PR/8/34 H1N1 serum per mL (#03/242; NIBSC; Hertfordshire, England). Measurements are reported in μg_HA_ per mL. The RSD of technical duplicates was ≤ 4.9%.

### PCR measurements

To analyze the genomic vRNA of virus particles, PCR-based methods were used. The vRNA in the supernatant samples was purified using the NucleoSpin® RNA virus kit (Macherey-Nagel, 740,956) according to manufacturer’s instructions. To identify vRNAs containing a deletion (indicating the presence of DIPs), a segment-specific RT-PCR was used (section “Segment-specific RT-PCR”). For quantification of Seg5, Seg8, and the deleted DI244 vRNA, a real-time RT-qPCR was used (section “Real-time RT-qPCR”).

#### Segment-specific RT-PCR

For reverse transcription (RT)-PCR, a previously described method was used [78]. In short, RT of RNA samples (isolated vRNAs of released virus particles) was carried out as a single reaction using a universal primer that hybridizes to the conserved 3′ region of all eight genome segments [79]. For the following PCR, individual segment specific primers were used. Amplified PCR products were visualized by agarose gel electrophoresis, and investigated for the presence of short PCR products, indicating DI vRNAs.

#### Real-time RT-qPCR

For real-time RT-qPCR, a method described previously [80] was used that allows gene-specific quantification of individual IAV vRNA segments. In vitro generation of reference standards, RT, real-time qPCR, and absolute quantification of vRNA levels are described elsewhere [17, 81]. To allow quantification of DI244 vRNA, a primer that binds across the junction region of DI244 was used [36]. The RSD of technical quadruplicates was ≤ 52.5%.

### Differential centrifugal sedimentation

DCS was used to determine the particle size distribution of DI244 material before and after purification, as reported previously [82] . For this, a CPS DC24000 UHR disc centrifuge (CPS Instruments Inc.; Los Angeles, USA) operated at 24,000 rpm with a 4–16% (m/v) sucrose gradient in PBS was used. The gradient was formed by nine 1.6 mL steps with a different sucrose concentration each (16%, 14.5%, 13%, 11.5%, 10%, 8.5%, 7%, 5.5%, and 4% sucrose (m/v)) resulting in a total volume of 14.4 mL. For gradient quality evaluation, a 239-nm particle standard (0.3–0.5% solid content, polyvinyl chloride, CPS Instruments Inc.; Los Angeles, USA) was injected directly after gradient generation. After 10 min of equilibration, another injection of a 239-nm particle standard followed for measurement calibration. Finally, 100 μL of the undiluted sample was injected for the size distribution measurements. Densities were 1.072 g/cm^3^ for the gradient buffer, 1.385 g/cm^3^ for the calibration particles, and 1.180 g/cm^3^ for IAV. The particle size distribution is shown as normalized weight average (in percent) against the apparent hydrodynamic diameter.

### Quantitation of total protein and host cell dsDNA

Total protein was measured using a Bradford BioRad assay (#5000006; BioRad Laboratories; Hercules, USA). The calibration curve was made with bovine serum albumin (#A3912; Sigma-Aldrich Chemie GmbH; Munich, Germany) in the range of 5–40 μg/mL and had a limit of detection of 0.4 μg/mL. The RSD of technical triplicates was ≤ 6.3%.

The concentration of host cell dsDNA was estimated with a Quant-iTTM PicoGreen® assay (# P7581; Life Technologies GmbH; Darmstadt, Germany). The standard curve was made with λ DNA (#D1501; Promega; Madison, USA) in the range of 4–250 ng/mL and had a limit of detection of 1.6 ng/mL. The RSD of technical duplicates was ≤ 2.1%.

## Supplementary Information

**Additional file 1: Figure S1.** Maximum specific growth rates of suspension MDCK cells. **Figure S2.** Interfering efficacy of DI244 material produced at different MODIPs. **Figure S3.** Interfering efficacy of DI244 material produced at MODIP 1E−2 in different cultivation vessels. **Table S1.** Overview on DI244 titers reported for shake flask cultivations in chapter 3.

## Data Availability

The datasets generated and analyzed during the current study are available in the Mendeley repository [83]. Data generated and analyzed during this study are also included in this published article and its additional files. The Supplementary Material for this article is attached. Cell lines and mouse strains will be made available upon request.
