# Supplementary material for: Cell culture-based production and in vivo characterization of purely clonal defective interfering influenza virus particles
Source: BMC Biol. 2021 May 3;19:91. doi: 10.1186/s12915-021-01020-5 (PMC8091782; doi:10.1186/s12915-021-01020-5)
Supplement: Supplementary file 1 — Additional file 1: Figure S1. Maximum specific growth rates of suspension MDCK cells. Figure S2. Interfering efficacy of DI244 material produced at different MODIPs. Figure S3. Interfering efficacy of DI244 material produced at MODIP 1E−2 in different cultivation vessels. Table S1. Overview on DI244 titers reported for shake flask cultivations in chapter 3. [file 12915_2021_1020_MOESM1_ESM.docx]

***Additional file 1***


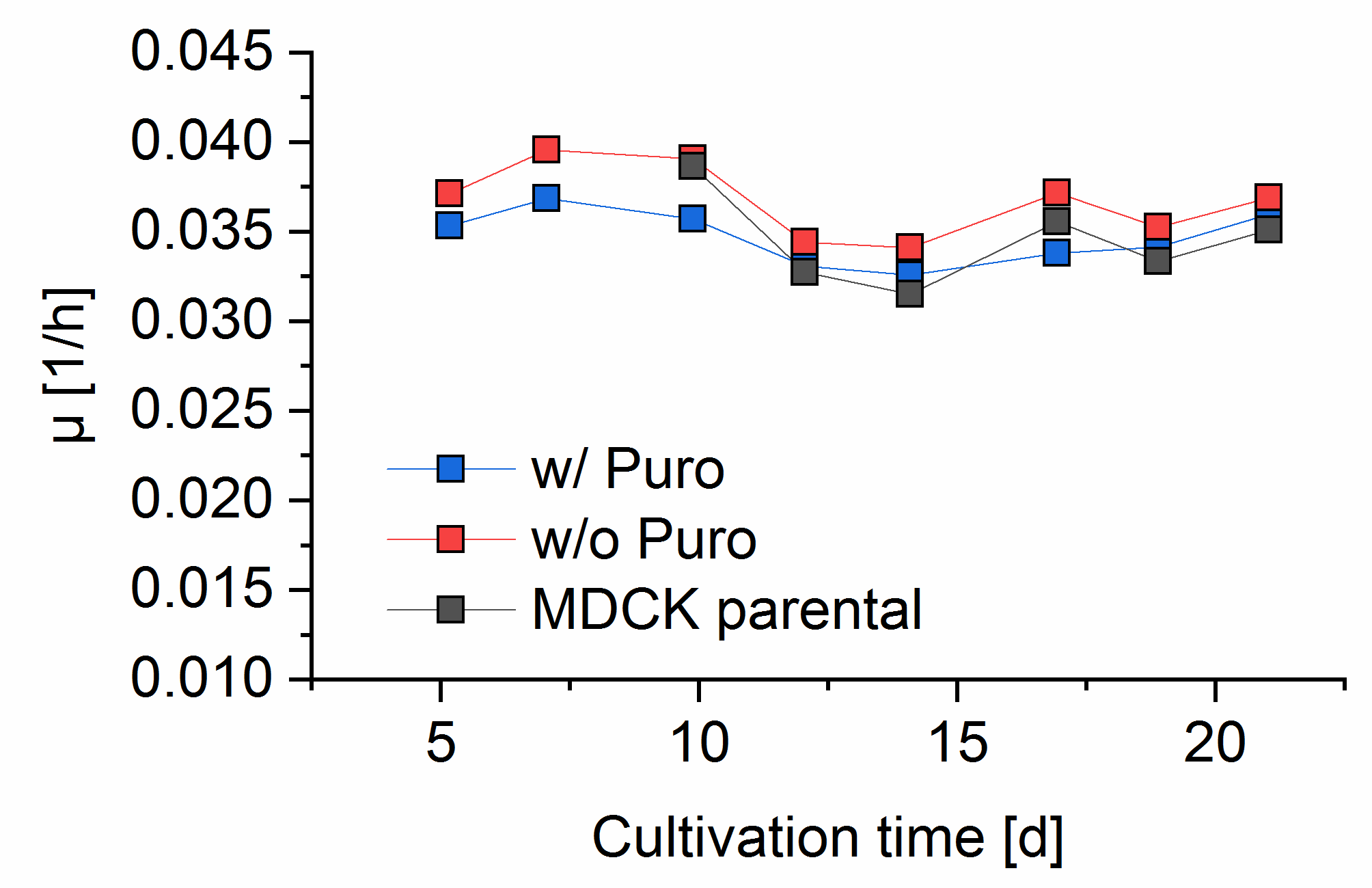


Figure S1: Maximum specific growth rates of suspension MDCK cells. Suspension MDCK-PB2(sus) cells were cultivated in chemically defined Xeno™ medium in shake flask with 50 mL working volume. Cells passaged every 2–3 days and specific growth rate was determined. Growth of MDCK-PB2(sus) cells with (w/ Puro) and without puromycin (w/o Puro) in Xeno™ medium was compared to growth of the parental MDCK cell line in Xeno™ medium without puromycin.


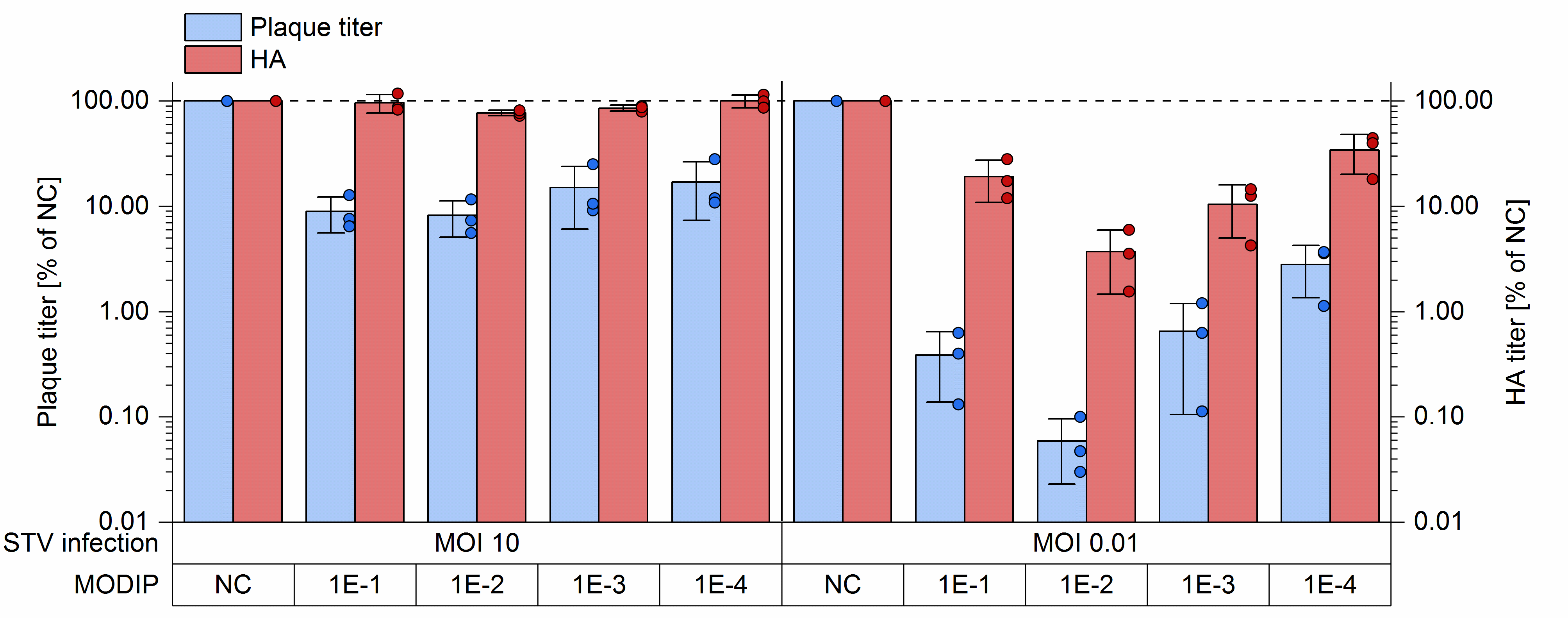


Figure S2: Interfering efficacy of DI244 material produced at different MODIPs. For the interference assay, parental adherent MDCK cells were infected with STV at MOIs of 10, or 0.01 and co-infected with DI244 material (125 µL), produced at an MODIP ranging from 1E−1 to 1E−4, or medium as negative control (NC). The supernatant was sampled 16 hpi (STV MOI 10) or 24 hpi (STV MOI 0.01). Infectious virus titers were quantified by plaque assay (parental adherent MDCK cell). The total amount of virus particles was determined by hemagglutination assay. The interference assay was performed in independent experiments (n = 3). The plaque and HA titer was normalized to the corresponding NC. The error bars indicate one standard deviation.


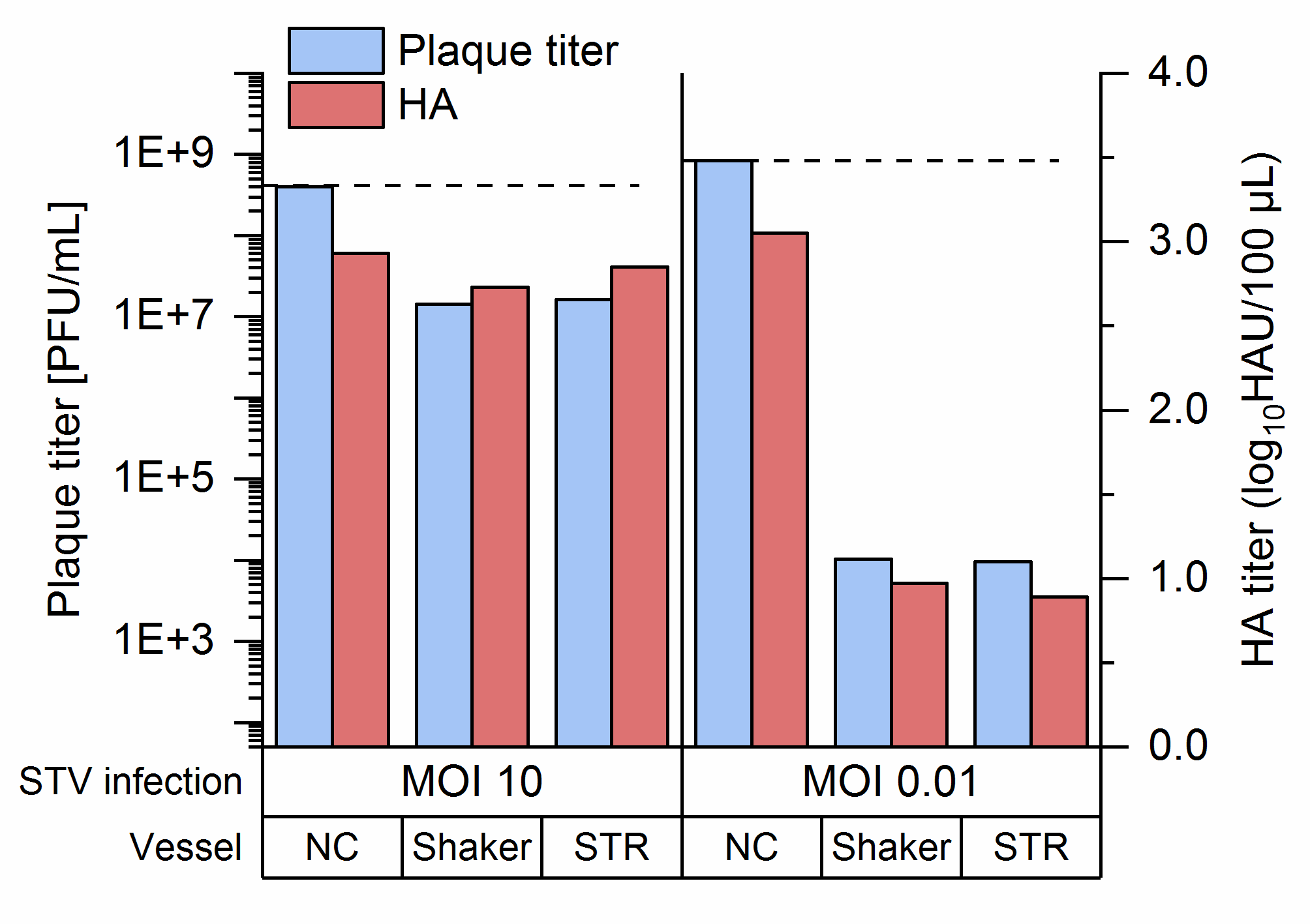


Figure S3: Interfering efficacy of DI244 material produced at MODIP 1E−2 in different cultivation vessels. For the interference assay, parental adherent MDCK cells were infected with STV at an MOI of 10, or 0.01 and co-infected with DI244 material (125 µL) or medium as negative control (NC). The supernatant was sampled 16 hpi (STV MOI 10) or 24 hpi (STV MOI 0.01). Infectious virus titers were quantified by plaque assay (parental adherent MDCK cells). The total amount of virus particles was determined by HA assay. The interference assay was performed for a single experiment using a preparation of DI244 produced with MODIP 1E−2 either in shake flask (Fig. 1) or in STR (Fig. 4).

Table S1: Overview on DI244 titers reported for shake flask cultivations in chapter 3. Harvest time varied from 18 to 30 hpi depending on MODIP.

| Production MODIP | Assay | Chapter 3.1 | Chapter 3.2 | Chapter 3.4/5 |
| --- | --- | --- | --- | --- |
| 1E−1 | HA assay (log_10_ HA units/100 µL) | 2.40 | 2.28 |  |
|  | DIP titer (PFU/mL) | 2.0E+7 | 2.5E+7 |  |
|  | Real-time RT-qPCR (DI244 vRNA copies/mL) | 5.0E+9 | 7.7E+9 |  |
| 1E−2 | HA assay (log_10_ HA units/100 µL) | 2.41 | 2.31 | 2.64 |
|  | DIP titer (PFU/mL) | 1.0E+8 | 8.0E+7 | 1.35E+8 |
|  | Real-time RT-qPCR (DI244 vRNA copies/mL) | 3.0E+9 | 5.4E+9 | 4.9E+9 |
| 1E−3 | HA assay (log_10_ HA units/100 µL) | 2.48 | 2.42 |  |
|  | DIP titer (PFU/mL) | 1.1E+8 | 7.6E+7 |  |
|  | Real-time RT-qPCR (DI244 vRNA copies/mL) | 1.5E+9 | 1.2E+9 |  |
| 1E−4 | HA assay (log_10_ HA units/100 µL) | 2.57 | 2.50 |  |
|  | DIP titer (PFU/mL) | 7.4E+7 | 6.0E+7 |  |
|  | Real-time RT-qPCR (DI244 vRNA copies/mL) | 4.4E+8 | 3.6E+8 |  |
